# Urate‐lowering therapy alleviates atherosclerosis inflammatory response factors and neointimal lesions in a mouse model of induced carotid atherosclerosis

**DOI:** 10.1111/febs.14768

**Published:** 2019-02-09

**Authors:** Jie Lu, Mingshu Sun, Xinjiang Wu, Xuan Yuan, Zhen Liu, Xiaojie Qu, Xiaopeng Ji, Tony R. Merriman, Changgui Li

**Affiliations:** ^1^ Institute of Metabolic Diseases Qingdao University China; ^2^ Shandong Provincial Key Laboratory of Metabolic Diseases and Qingdao Key Laboratory of Gout The Affiliated Hospital of Qingdao University China; ^3^ Department of Endocrinology and Metabolic Diseases The Affiliated Hospital of Qingdao University China; ^4^ Department of Rheumatology and Clinical Immunology The Affiliated Hospital of Qingdao University China; ^5^ Department of Biochemistry University of Otago Dunedin New Zealand

**Keywords:** allopurinol, animal model, atherosclerosis, reactive oxygen species, urate

## Abstract

Hyperuricemia (HU) is a cause of gout. Clinical studies show a link between HU and cardiovascular disease. However, the role of soluble serum urate (SU) on atherosclerosis development remains elusive. We aimed to use a new HU mouse model [*Uricase/Uox* knockout (KO)] to further investigate the relationship between HU and atherosclerosis. A mouse model by perivascular collar placement of induced carotid atherosclerosis was established in male *Uox‐* KO mice. The *Uox‐* KO mice had elevated SU levels and enhanced levels of atherosclerosis inflammatory response proteins. In contrast, *Uox‐* KO mice with carotid atherosclerosis showed severe neointimal changes in histology staining consistent with increases in intimal area and increases in proliferating cell nuclear antigen (PCNA)‐ and F4/80‐positive cells. Allopurinol reduced neointimal areas induced by the perivascular collar in hyperuricemic mice, accompanied by decreased expression of PCNA‐ and F4/80‐positive cells. Urate‐lowering treatment alleviated atherosclerosis inflammatory response factors and reactive oxygen species (ROS) intensities in both collar placement *Uox‐* KO mice and urate‐stimulated human umbilical vein endothelial cells (HUVECs)*. In vitro* results using HUVECs showed ROS was induced by urate and ROS induction was abrogated using antioxidants. These data demonstrate that urate *per se* does not trigger atherosclerosis intima lesions in male mice. Urate worsens carotid neointimal lesions induced by the perivascular collar and urate‐lowering therapy partially abrogates the effects. The current study warrants clinical studies on the possible benefits of urate‐lowering therapy in atherosclerosis patients with HU.

## Graphical abstract

Clinical studies show a link between hyperuricemia (HU) and cardiovascular disease. However, the role of soluble urate on atherosclerosis development remains unclear. A mouse model of induced carotid atherosclerosis was established in a spontaneous HU mouse with the uricase gene inactivated. This study demonstrates that urate worsens carotid neointimal lesions induced by the perivascular collar and that urate‐lowering therapy partially abrogates the effects.

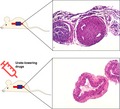

**Abbreviations**
BUN: blood urea nitrogen
CKD: chronic kidney disease
CVD: cardiovascular disease
HDL‐C: high‐density lipoprotein cholesterol
HU: hyperuricemia
HUVECs: human umbilical vein endothelial cells
ICAM‐1: intercellular adhesion molecule‐1
KO: knockout
LDL‐C: low‐density lipoprotein cholesterol
MCP‐1: monocyte chemoattractant protein‐1
NAC: *N*‐acetylcysteine
PCNA: proliferating cell nuclear antigen
ROS: reactive oxygen species
SU: serum urate
TC: total cholesterol
TG: triglyceride
ULT: urate‐lowering treatment
*Uox*: uricase/urate oxidase
VCAM‐1: vascular cell adhesion molecule‐1
WT: wild type
XO: xanthine oxidase

## Introduction

Cardiovascular disease (CVD) is the leading cause of death world‐wide 1. Atherosclerosis is one of the major CVDs characterized by focal intimal thickening and ultimately luminal obstruction induced by fibro‐proliferation and an inflammatory process mediated by cytokine production and vascular regulatory mechanisms 2. Neointimal formation happens at the early stage of atherosclerosis which is a complex process initiated by the damage of endothelial cells and exposure of vascular smooth muscle cells to circulating blood elements 3. Hyperuricemia (HU) causes gout and has been implicated in hypertension and atherosclerosis in humans 4. Epidemiological studies have associated HU with atherosclerotic vascular diseases 5, and the level of serum urate (SU) can predict cardiovascular outcomes and mortalities in both sexes 6. HU is also a risk factor for hypertension, which is a potent contributor of atherosclerosis 7. A meta‐analysis of 18 prospective cohort studies, including data from more than 55 000 patients, showed an increased risk of incident hypertension in subjects with HU, and the overall risk increased by 13% per 1 mg·dL^−1^ increase in SU 8. More recently, a retrospective cohort study demonstrated that HU plays a significant role in the progression from prehypertension to hypertension with a 35% increasing ratio of hypertension risk in men 9. These findings have prompted a growing research interest on the possible benefits of urate‐lowering treatment (ULT) in cardiovascular diseases. However, it has not been definitively established whether urate is merely a marker or a causal agent of CVD, or whether ULT affects outcomes.

Our previous data demonstrated elevated blood pressure in female but not male mice suggesting distinct mechanism accounting for the vascular phenotypes between males and females 10. Given HU is more important for males in term of prevalence, we wanted to ask: (a) does HU in males induce endothelial injury at micro level if not high blood pressure at macro level; (b) if not, does HU worsen existing neointimal lesions induced using established atherosclerosis model. In this study, males were employed to address these questions.

## Results

### Soluble urate does not induce atherosclerotic phenotypes

Serum urate levels in male uricase/urate oxidase (*Uox*)‐KO mice were almost three‐fold increased compared to wild‐type (WT) counterparts (563.9 μmol·L^−1^ ± 16.9 vs 176.3 μmol·L^−1^ ± 6.7, *P* < 0.001; Table 1). Compared with WT controls, blood urea nitrogen (BUN) and serum creatinine were significantly elevated in *Uox*‐KO mice (Table 1). No apparent changes occurred in the fasting glucose level of male *Uox*‐KO mice compared with WT mice (Table 1). Neither did lipid profiles change [total cholesterol (TC), high‐density lipoprotein cholesterol (HDL‐C) and low‐density lipoprotein cholesterol (LDL‐C); Table 1].

**Table 1:** Blood biochemistry in WT and *Uox*‐KO mice. Biochemical indicators in WT and *Uox*‐KO mice (*n* = 8 per group, males, 16 weeks of age). Data were shown as mean ± SEM. Differences between groups were analyzed by the student's *t* test and one‐way analysis of variance followed by Newman–Keuls multiple comparison test

	WT	KO	*P* value	WT‐collared	KO‐collared	*P* value	KO‐collared + allopurinol	*P* value (compared to KO‐collared)
Uric acid (μmol·L^−1^)	176.3 ± 6.7	563.9 ± 16.9	< 0.001	187.5 ± 4.9	573.0 ± 16.4	< 0.001	325.8 ± 9.2	< 0.001
BUN (mmol·L^−1^)	4.47 ± 0.16	10.04 ± 0.35	< 0.001	5.19 ± 0.28	10.42 ± 0.42	< 0.001	7.91 ± 0.63	0.005
Creatinine (μmol·L^−1^)	16.53 ± 1.67	23.63 ± 2.18	0.04	15.36 ± 1.83	30.5 ± 0.97	< 0.001	21.77 ± 2.47	0.0047
Fasting glucose (mmol·L^−1^)	6.0 ± 0.2	6.3 ± 0.2	0.25	6.1 ± 0.2	6.2 ± 0.2	0.71	6.4 ± 0.3	0.6
TC (mmol·L^−1^)	3.36 ± 0.12	3.67 ± 0.13	0.1	3.59 ± 0.08	3.85 ± 0.12	0.08	3.62 ± 0.14	0.23
TGs (mmol·L^−1^)	1.49 ± 0.16	1.13 ± 0.03	0.03	1.35 ± 0.07	1.23 ± 0.03	0.13	1.27 ± 0.06	0.62
HDL cholesterol (mmol·L^−1^)	1.61 ± 0.13	1.74 ± 0.05	0.35	1.60 ± 0.05	1.80 ± 0.08	0.06	1.59 ± 0.13	0.21
LDL cholesterol (mmol·L^−1^)	0.49 ± 0.07	0.55 ± 0.04	0.44	0.48 ± 0.06	0.55 ± 0.06	0.43	0.50 ± 0.03	0.44

**Figure 1:**
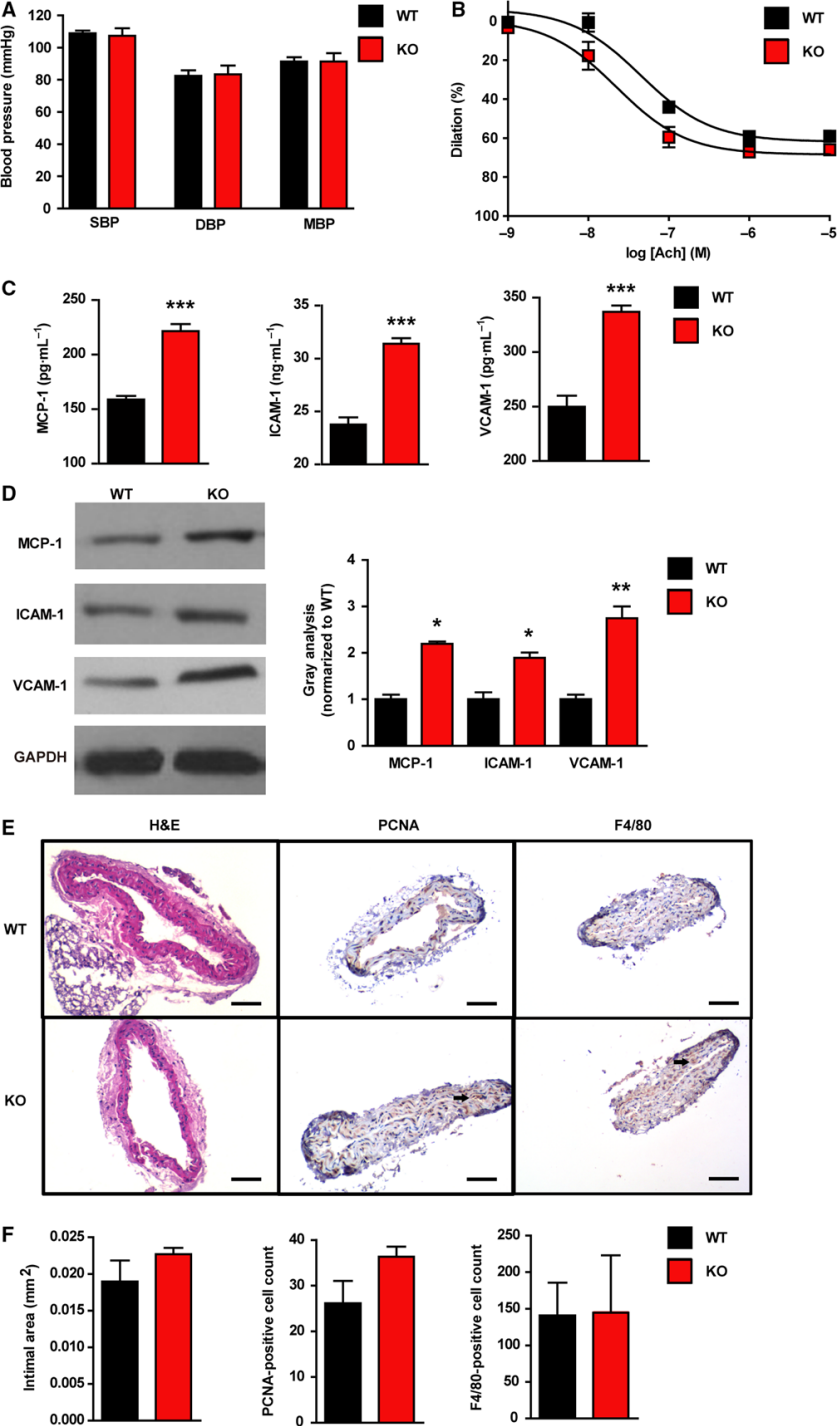
Soluble urate does not induce atherosclerosis phenotypes. (A) Blood pressure was measured by the CODA programmable non‐invasive tail‐cuff sphygmomanometer. Mice underwent an acclimation period of seven consecutive days to the sphygmomanometer before experiments. SBP, DBP and MBP in non‐fasting 16‐week‐old WT and *Uox*‐KO mice (males, *n* = 10, 7). (B) Isolated aortic rings from 16‐week‐old WT and *Uox*‐KO mice (males, *n* = 3) were precontracted with 0.1 μmol·L^−1^ noradrenaline after an equilibration period of 60 min. Dilation at each acetylcholine concentration (0.001, 0.01, 0.1, 1.0, 10 μmol·L^−1^) was measured and expressed as the percentage response to noradrenaline. (C) Protein levels of MCP‐1, ICAM‐1, and VCAM‐1 in serum of 16‐week‐old WT and *Uox*‐KO mice (males, *n* = 6) detected by ELISA. (D) Protein levels of MCP‐1, ICAM‐1, and VCAM‐1 in carotid arteries of 16‐week‐old WT and *Uox*‐KO mice determined by three independent western blotting experiments (males, *n* = 3). (E, F) Carotid arteries were removed and fixed in formalin followed by paraffin‐embedding of 5 μm serial sections. Pathological assessment in WT and *Uox*‐KO mice (males, *n* = 6) quantified by intimal area, PCNA‐ and F4/80‐ positive cell counts. Scale bars = 50 μm. Error bars represent SEM. *P < 0.05, **P < 0.01, ***P < 0.001 vs WT (student's *t*‐test).

To evaluate the effects of urate on the cardiovascular system, blood pressure, endothelium‐dependent vasodilatation and cardiac function were tested. Systolic blood pressure (SBP), diastolic blood pressure (DBP) and mean blood pressure (MBP) exhibited no difference between male *Uox*‐KO mice and WT controls (Fig. 1A). Neither did aortae dilation nor cardiovascular function change (Fig. 1B; Table 2). This does not support a direct role of urate in the control of blood pressure, vasodilation and cardiac function in males. As shown in Fig. 1C,D pathogenic proteins in atherosclerosis were up‐regulated in both plasma and carotid lesions – monocyte chemoattractant protein (MCP‐1), intercellular adhesion molecule‐1 (ICAM‐1), and vascular cell adhesion molecule‐1 (VCAM‐1; *P* < 0.05). However, *Uox*‐KO mice did not present an atherosclerotic phenotype, showing no changes in intimal areas, proliferation (proxied by PCNA‐positive cells) and inflammation (proxied by F4/80‐positive cells) in Fig. 1E,F.

**Table 2:** The results of transthoracic ultrasound. No significant difference was observed between male *Uox*‐KO and WT counterparts (*n* = 6, 16 weeks of age). LVAWd: end‐diastolic left ventricular anterior wall thickness; LVAWs: end‐systolic left ventricular anterior wall thickness; LVIDd: end‐diastolic left ventricular inner diameter; LVIDs: end‐systolic left ventricular inner diameter; LVPWd: end‐dastolic left ventricular posterior wall thickness; LVPWs: end‐systolic left ventricular posterior wall thickness; EF: ejection fraction; FS: fractional shortening; LV Mass AW: left ventricle mass. Differences between groups were analyzed by the student's *t* test

	WT (*n* = 6)		KO (*n* = 6)		*P*
	Mean	SEM	Mean	SEM	
LVAW; d (mm)	0.88	0.05	0.84	0.05	0.57
LVAW; s (mm)	1.20	0.09	1.14	0.08	0.60
LVID; d (mm)	3.43	0.07	3.72	0.13	0.08
LVID; s (mm)	2.48	0.14	2.73	0.15	0.26
LVPW; d (mm)	0.81	0.04	0.85	0.07	0.66
LVPW; s (mm)	1.05	0.07	1.11	0.09	0.62
EF (%)	54.38	4.40	52.04	4.93	0.73
FS (%)	27.77	2.81	26.52	3.01	0.77
LV mass AW (mg)	98.18	4.74	111.40	5.21	0.09
LV mass AW (corrected; mg)	78.54	3.79	89.12	4.16	0.09
LV Vol; d (μL)	48.47	2.43	59.50	4.91	0.07
LV Vol; s (μL)	22.61	3.11	28.59	3.89	0.26
Stroke volume (μL)	25.86	1.09	30.92	3.98	0.25
Cardiac output (mL·min^−1^)	9.71	0.74	10.17	1.15	0.74
Heart rate (b.p.m.)	375	22	342	35	0.50

### Hyperuricemia accelerates carotid neointimal lesions with collar placement

**Figure 2:**
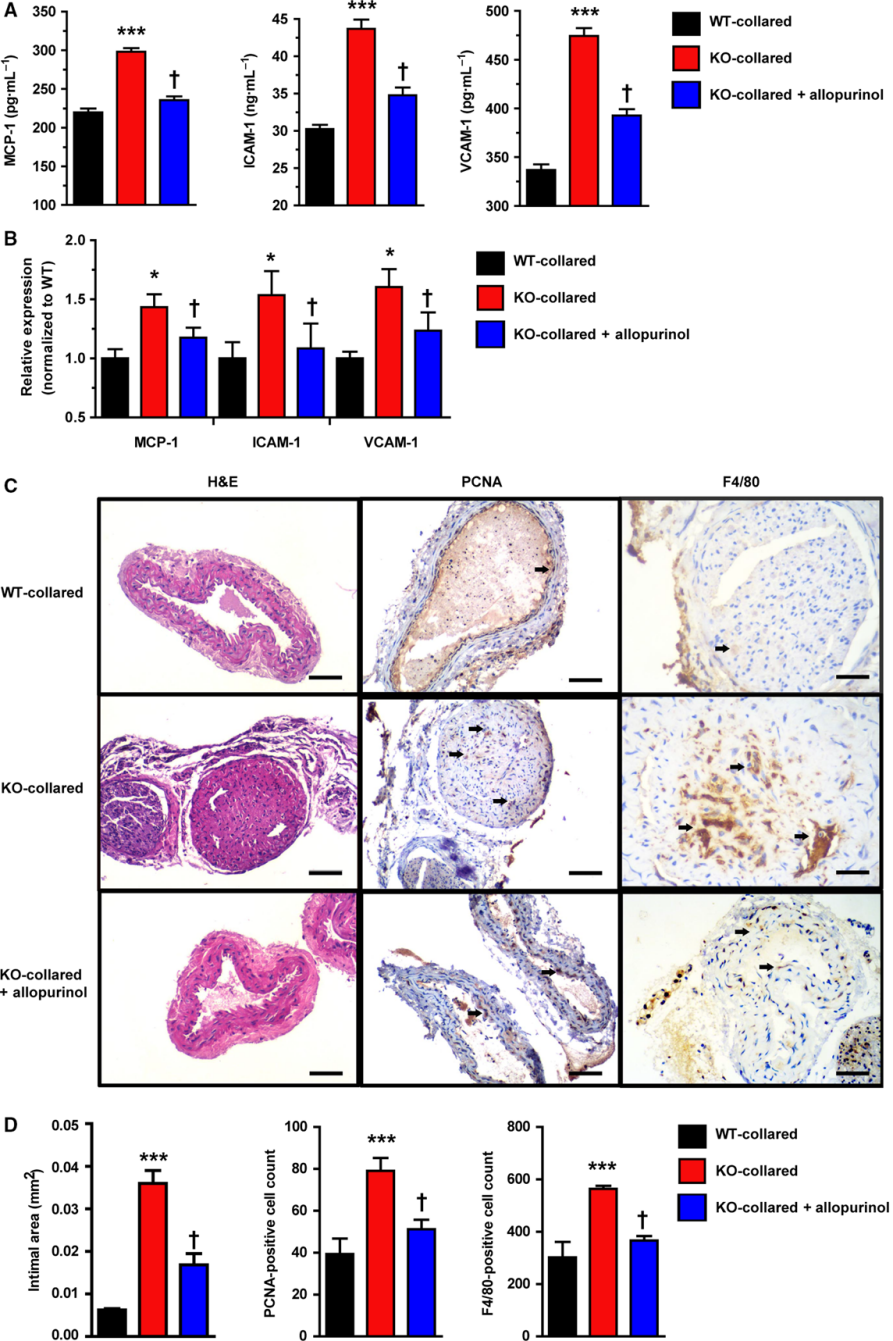
HU accelerates carotid neointimal lesions with collar placement. A perivascular carotid collar placement model was generated with non‐occlusive silastic collars in 10‐week‐old mice. Four weeks before surgery, the animals received HF, HC western‐type diets [21% (wt/wt) fat and 0.15% cholesterol]. Mice were gavaged for a 10‐week allopurinol treatment (100 mg·kg^−1^). Shown are effects of collar induction and allopurinol treatment on MCP‐1, ICAM‐1, and VCAM‐1 levels in (A) plasma (*n* = 6) and (B) carotid tissues (males, *n* = 6), and carotid morphology, proliferation and inflammation represented by intimal area, PCNA‐ and F4/80‐ positive cell counts in (C, D) (males, *n* = 6), respectively. Scale bars = 50 μm. Error bars represent SEM. *P < 0.05, ***P < 0.001 vs WT control and ^†^ *P* < 0.05 vs untreated *Uox*‐KO mice (student's *t* test and one‐way analysis of variance followed by Newman–Keuls multiple comparison test).

We next addressed the questions: is the effect of urate evident only once a stress is given, and does ULT affect the atherosclerosis pathogenesis? This perivascular carotid collar model along with western‐type diet represents a useful complementary model in which neointimal lesions show up at the early‐stage of atherosclerosis. With collar induction HU makes neointimal lesions worse, indicated by the increases in intimal area, more PCNA‐ and F4/80‐ positive cells in the carotid artery compared with WT collared mice (Fig. 2C,D). Administration of allopurinol (100 mg·kg^−1^), a xanthine oxidase (XO) inhibitor and urate‐lowering drug, significantly alleviated the intimal phenotypes including intimal area, PCNA‐positive cell counts and F4/80‐positive cell counts. Allopurinol also alleviated both SU and renal functions indicated by BUN and creatinine in *Uox*‐KO collared mice compared to WT collared mice (Table 1). Consistently, allopurinol decreased expression of MCP‐1, as well as ICAM‐1 and VCAM‐1, in both plasma and carotid tissues determined by ELISA and RT‐PCR, respectively (Fig. 2A,B). The down‐regulation of MCP‐1, ICAM‐1 and VCAM‐1 in carotid tissue indicated that inflammation was improved by ULT. Echocardiographic analysis of heart rate, diastolic left ventricular internal diameter and ejection fraction exhibited no difference between collared *Uox*‐KO mice and WT controls (data not shown).

### Hyperuricemia elevates ROS in carotid artery *in vivo*

**Figure 3:**
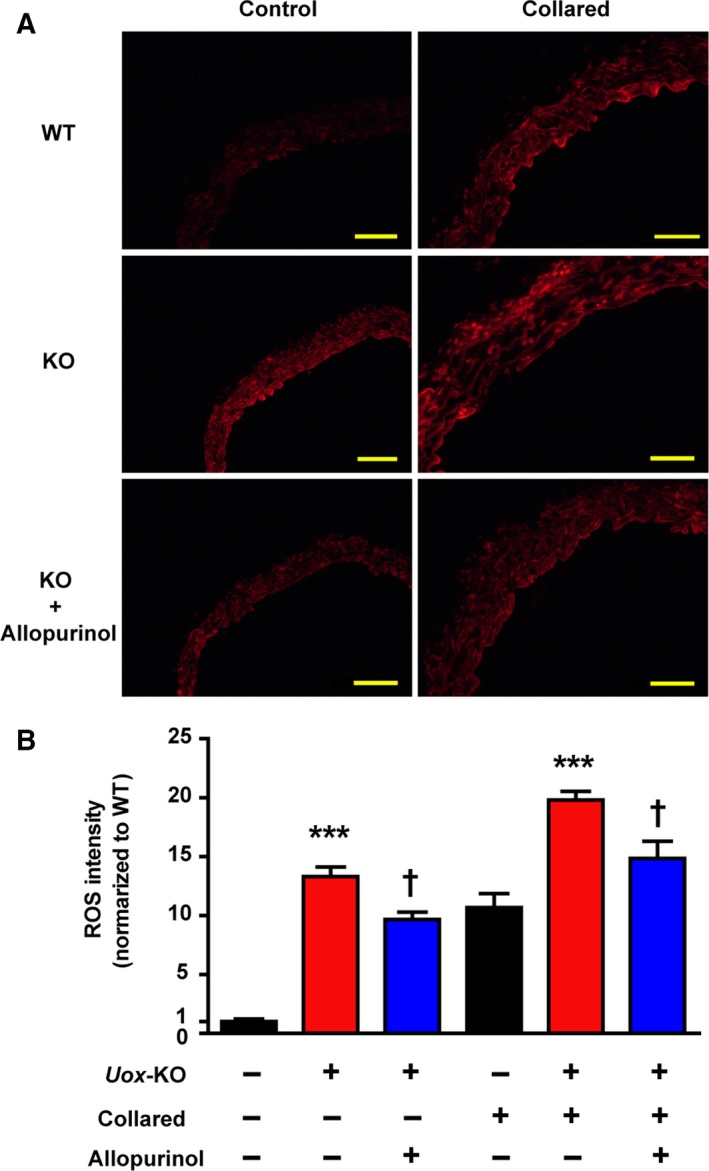
HU elevates ROS in carotid artery *in vivo*. Carotid arteries were incubated with 2 μmol·L^−1^ DHE fluorescence probe for 30 min at 37 °C in the dark to measure ROS levels. (A) Representative images of fluorescent dye DHE staining from carotid artery of WT and *Uox*‐KO mice with or without collar placement are shown (males, *n* = 6). (B) Effects of 8‐week allopurinol treatment (100 mg·kg^−1^) on ROS were quantified. Scale bars = 50 μm. Error bars represent SEM. ***P < 0.001 vs WT control. ^†^ *P* < 0.05 vs untreated *Uox*‐KO mice (student's *t* test and one‐way analysis of variance followed by Newman–Keuls multiple comparison test).

Given reactive oxygen species (ROS) plays a putative role in the pathogenesis of atherosclerosis, we tested the ROS intensity by fluorescent dye dihydroethidium (DHE) staining in the carotid artery of WT and *Uox*‐KO mice. *Uox*‐KO mice had elevated ROS levels compared with WT, with or without collar placement (Fig 3A, 3B). ROS intensities were reduced in *Uox*‐KO mice with 100 mg·kg^−1^ allopurinol treatment for 10 weeks vs WT controls in the presence or absence of collar placement (Fig 3A,B), implying that urate imposes additional oxidative stress which may accelerate atherosclerosis development.

### Soluble urate induces ROS enhancement *in vitro*

**Figure 4:**
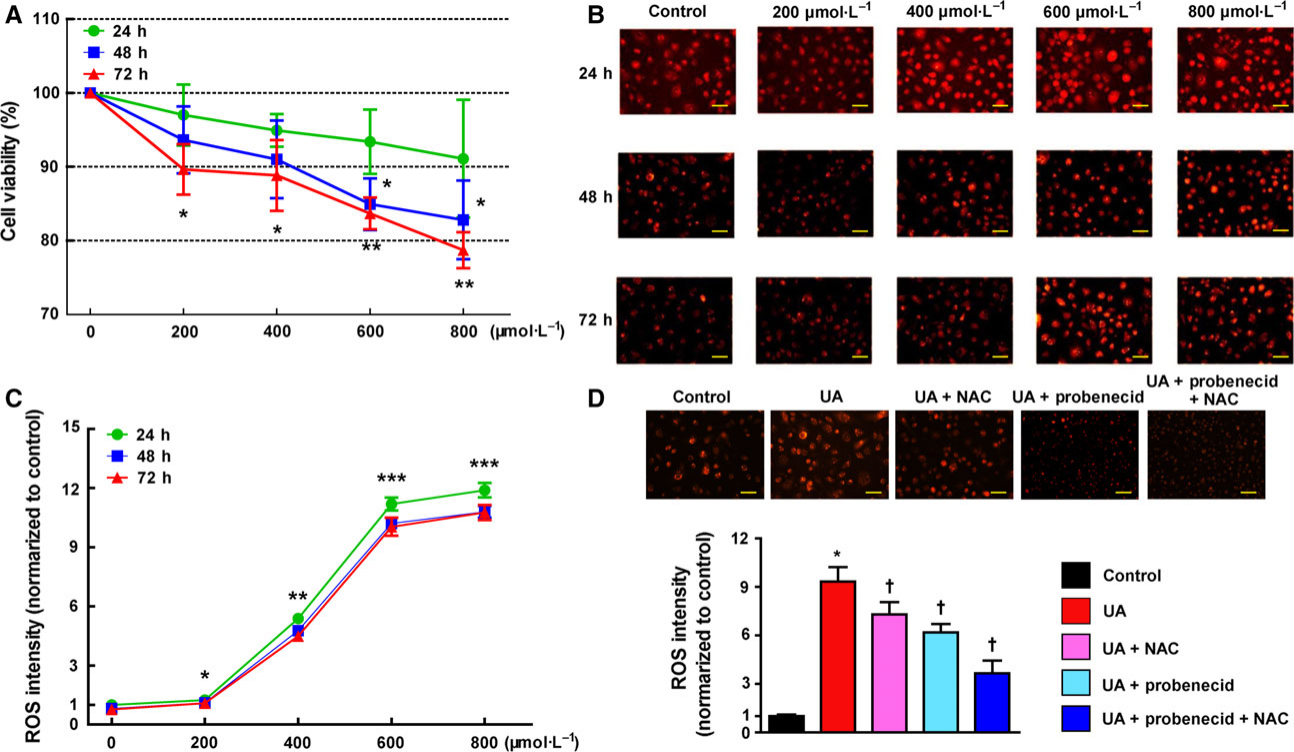
Soluble urate induces ROS enhancement *in vitro*. (A) HUVECs (10^5^/well) viability measurement co‐incubated with soluble urate (200, 400, and 800 μmol·L^−1^) at 24, 48 and 72 h. (B, C) Representative images of fluorescent dye DHE staining from soluble urate stimulated HUVECs and quantified ROS levels. (D) DHE staining on soluble urate (800 μmol·L^−1^, 48 h) stimulated HUVECs after 12 h pre‐incubation with 1 mmol·L^−1^ probenecid or 10 μmol·L^−1^ *N*‐acetyl‐l‐cysteine (NAC) or both. Scale bars = 20 μm. Error bars represent SEM. *P < 0.05, **P < 0.01, ***P < 0.001 vs control, ^†^ *P* < 0.05 vs UA group (student's *t* test and one‐way analysis of variance followed by Newman–Keuls multiple comparison test).

**Figure 5:**
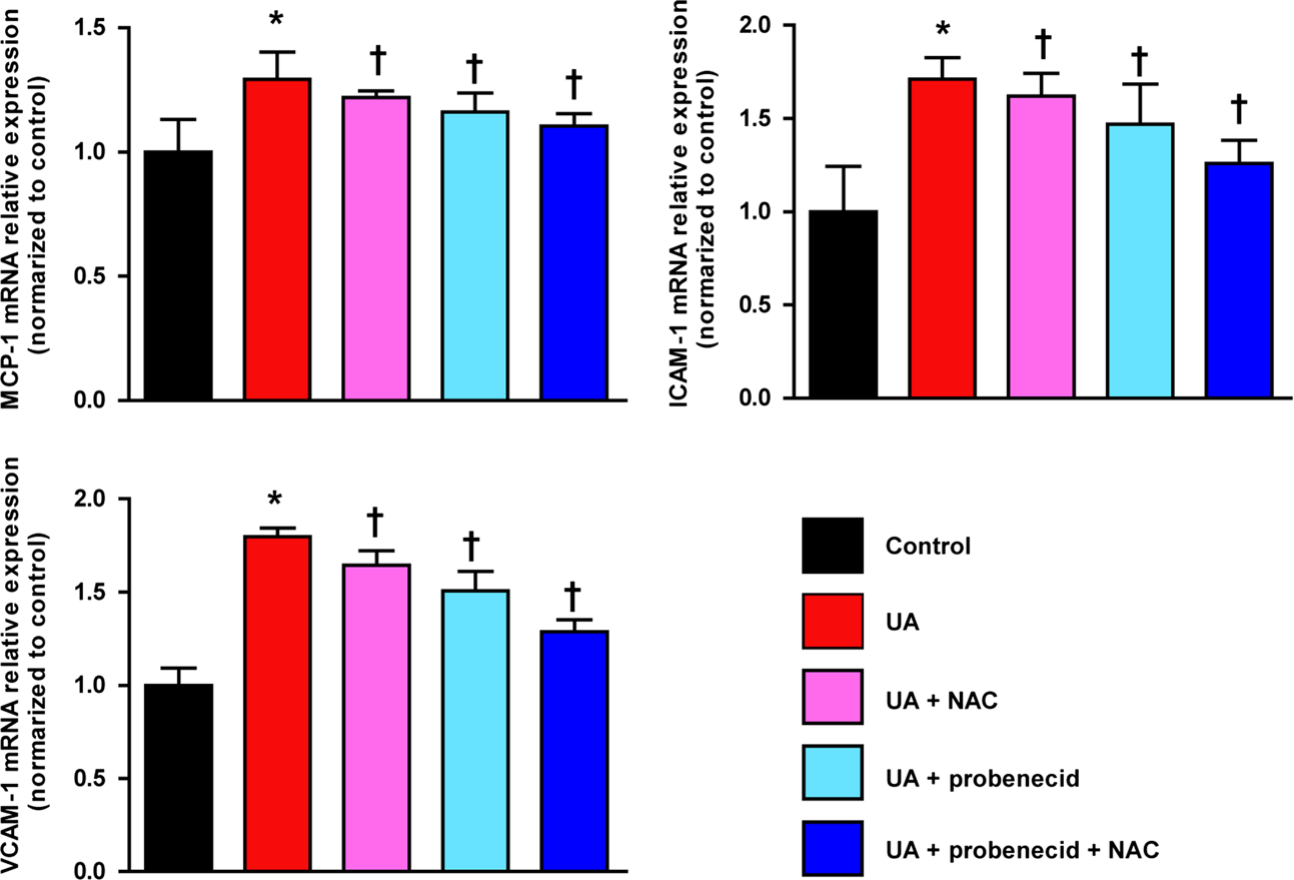
ULT alleviates atherosclerosis inflammatory response factors *in vitro*. Soluble urate ‐stimulated HUVECs (800 μmol·L^−1^, 48 h) after 12 h pre‐incubation with 1 mmol·L^−1^ probenecid or 10 μmol·L^−1^ NAC or both. Relative mRNA expression of MCP‐1, ICAM‐1, and VCAM‐1 in HUVECs was estimated. Error bars represent SEM. *P < 0.05 vs control, ^†^ *P* < 0.05 vs UA group (student's *t* test and one‐way analysis of variance followed by Newman–Keuls multiple comparison test).

Human umbilical vein endothelial cell (HUVEC) (10^5^/well) viability was measured when co‐incubated with soluble urate (200, 400, 600 and 800 μmol·L^−1^) at 24, 48 and 72 h. As shown in Fig. 4A–C, soluble urate decreased cell viability in a dose‐dependent and time‐dependent manner and elevated ROS levels – for example with 800 μmol·L^−1^ urate, ROS intensity was almost 10‐fold increased. ROS levels were extenuated by pre‐incubation for 12 h with probenecid (1 mmol·L^−1^), an organic anion transport inhibitor, or *N*‐acetylcysteine (NAC; 10 μmol·L^−1^), a ROS scavenger, or with both (Fig. 4D). Relative mRNA expression of MCP‐1, ICAM‐1 and VCAM‐1 in soluble urate (800 μmol·L^−1^, 48 h)‐treated HUVECs was significantly higher than controls and this was lessened by pre‐incubation with probenecid or NAC or both (Fig. 5).

## Discussion

The presence of hepatic *Uox* is the reason that rodents have lower SU compared to humans 11. When *Uox* is knocked out, mice develop spontaneous HU similar to the SU level of humans 10. Based on this HU mouse model, we further generated an atherosclerosis model with right carotid artery peri‐collar placement and a western‐type diet. Using this model we showed that, because *Uox*‐KO mice did not present an atherosclerotic phenotype despite of facts that inflammatory response and ROS indeed were induced in *Uox*‐KO mice. However, HU worsened the development of neointimal lesions through *in vivo* ROS enhancement.

Epidemiological studies have detected an association between HU and hypertension, while evidence for causation, that includes data from Mendelian randomization studies, is limited and inconclusive 12, 13, 14. Hypertension is a risk factor in the pathogenesis of atherosclerosis. Here, we describe the cardiovascular characteristics in *Uox*‐KO male mice, which exhibit no signs of heart dysfunction, heart morphology alteration or blood pressure change. This is consistent with data from another HU mouse model with liver *Glut9* deficiency 15. These data therefore do not support a direct causal role of urate on blood pressure in mice.

To the best of our knowledge, this study is the first to assess conducted dilation responses of aortae in the spontaneous HU mouse and showed that urate cannot induce experimentally significant changes of endothelium‐dependent vasodilatation. Indeed, when expressed as a function of the dilation, the conducted dilation of aortae in male *Uox*‐KO and WT mice was relatively similar. Interestingly, the endothelial relaxation dysfunction reported in aortae from low‐density lipoprotein receptor/apolipoprotein E (LDL/*ApoE*) double KO mice was in regions with significant lesions, and not in other regions or in aortae from the *ApoE* single KO mice where lesions were minimal 16. Similarly, even in diabetic *ApoE*‐KO mice, endothelial dysfunction was only reported in plaque‐prone regions of the aortae, while plaque‐resistant segments maintained a normal acetylcholine response 17. These pieces of evidence are consistent with our observation of no vasodilation dysfunction in our *Uox*‐KO mice compared with WT mice.

Our results show that spontaneous HU without any stress does not induce obvious atherosclerosis phenotypes. However, HU alone was sufficient to induce oxidative stress and enhance levels of atherosclerosis associated inflammatory cytokines indicating that HU is only a promoting factor rather than an initiator in atherosclerosis. HU may contribute to an activated atherosclerotic pathological process. MCP‐1, ICAM‐1 and VCAM‐1, crucial pathogenic elements in atherosclerosis, are up‐regulated in atherosclerotic lesions and influence growth factor production and medial smooth muscle cell migration 18, 19. HU enhanced MCP‐1, ICAM‐1 and VCAM‐1 as shown in serum level and protein expression, suggesting that these three molecules are also involved in a HU‐driven atherosclerosis promoting effect that would exacerbate the pathogenesis of atherosclerosis. The augmented response of MCP‐1, ICAM‐1 and VCAM‐1 to a given concentration of urate would stimulate downstream inflammation and thereby induce atherosclerosis progressing to a greater extent than controls *in vitro*. Increasing of intimal area due to smooth muscle cell movement and reproduction is an essential component of atherosclerosis, which can be indicated by proliferating cell nuclear antigen (PCNA) 20. Macrophages accumulate in atherosclerotic plaques, playing crucial roles in atherosclerotic immune responses 21. Significant increases in intimal areas, PCNA‐ and F4/80‐ positive cells in the carotid artery indicates that HU contributes to atherosclerosis, though no plague were found. Despite a few hundred systematic reviews, meta‐analyses, and Mendelian randomization studies exploring 136 unique health outcomes, convincing evidence of a clear causal role of urate in disease pathogenesis only exists for gout and nephrolithiasis 22. Urate is involved in a diverse array of biological functions, while possibly contributing to the pathogenesis of cardiovascular phenotypes, rendering it a pathogenic but not causal role 11. HU did associate with an increased risk of cardiovascular death only in participants with gout and existing cardiovascular disease 23, consistent with our experimental *in vivo* data.

Reactive oxygen species are another major but non‐specific mediator in the formation of atherosclerosis‐inducing endothelial dysfunction. It can reduce the bioavailability of nitric oxide, a potential anti‐atherosclerotic factor 24. Urate can lead to ROS enhancement which facilitates atherosclerosis by oxidative stress 25, which is consistent with our *in vivo* and *in vitro* data. Changes in ROS exhibited a similar trend as the change of neointimal lesions in collar‐induced *Uox*‐KO mice, which were partially rescued by ULT. This suggests that ROS may also contribute to urate‐induced atherosclerosis‐promoting effects. The effect of allopurinol (via the active metabolite oxypurinol) that inhibits XO activity and suppresses urate biosynthesis 26, also reduces ROS production by inhibiting XO. Other non‐XO effects of allopurinol include LDL oxidation prevention, heat shock protein expression inhibition and decreasing early changes in inflammation such as leukocyte activation by reducing adherence, rolling and extravasation 27. Given our data shows HU was sufficient to induce both ROS production and atherosclerosis associated inflammatory cytokines, worsened ROS level, inflammatory molecules and neointimal lesions observed in collared *Uox*‐KO mice could be effects due to an ‘additional action’ imposed by HU. Thus, all the neointimal lesions and associated inflammatory factors as well as ROS production were alleviated when ULT was present. HUVECs are wildly used cell models to investigate the mechanisms of vascular phenotypes, especially atherosclerosis 28, 29. HUVECs have urate transporter 1 (URAT1) on their apical‐surface, and probenecid is an effective inhibitor of URAT1. Pretreating HUVECs with probenecid will stop urate from entering inside the cells and its intracellular biologic behaviours efficaciously at the dose of 1 mmol·L^−1^ 30. *In vitro*, our study showed the same trend of ROS *in vivo*. ROS intensities were lowered with probenecid intervention. Combined with probenecid and NAC, the strongest ROS lowering effect was observed. Thus, the benefits of XO inhibitors such as allopurinol might rely on blocking the production of oxidants rather than on lowering urate 31. A random, double‐blind, crossover study also showed that the mechanism of improvement in endothelial function with high‐dose allopurinol lies in its ability to reduce vascular oxidative stress and not in urate reduction 32. Therefore, although our data exclude a direct causal role of urate *per se* on atherosclerosis in male *Uox*‐KO mice, further studies to address a potential role of XO activity on the cardiovascular function are warranted. Overall, this work reinforces the conclusion that urate accelerates pathogenesis of atherosclerosis and ROS lowering may bring the anti‐atherosclerotic effects.

It remains controversial as to whether asymptomatic HU should be treated for the purpose of improving cardiovascular outcomes 33. Stamp and Dalbeth 34 suggested that asymptomatic HU treatment needs to be cautiously considered, due to the limited data and the potential risks of treatment. Kok *et al*. 35 reported that allopurinol therapy in patients with gout does not yield beneficial cardiovascular outcomes. However, Kuwabara *et al*. 36 promoted the use of ULT for asymptomatic HU in a 5‐year Japanese cohort study with 13 201 subjects. ULT was associated to better outcomes in HU patients with cardiovascular diseases accompanied by benefits in endothelial dysfunction and systemic inflammation 37. A randomized controlled trial reported that allopurinol reduces central blood pressure and carotid intima‐media thickness progression at 1 year in patients with recent ischemic stroke and transient ischemic attack 38. A mouse study reported that allopurinol represents a potential novel strategy for preventing left ventricular remodelling and dysfunction after myocardial infarction 39. As well, significant anti‐atherosclerotic effects were seen in a HU study in *ApoE*‐KO mice 40. Consistently, our results suggest that ULT with allopurinol for hyperuricemic mice may improve neointimal lesions significantly.

Given multiple lines of evidence suggest patients with chronic kidney disease (CKD) are at an increased risk cardiovascular disease 41 and *Uox*‐KO mice develops renal failure 10, we could not rule out CKD is involved in the neointimal lesions in HU mice. Generally, CKD worsens classical risk factors including hypertension, dyslipidemia and malnutrition 42, 43, which are associated with increased thickness and stiffness of arteries independent of major confounders 44, 45. However, none of these phenotypes can be observed in the male *Uox*‐KO mice according to our previous report 10, which is distinct from 5/6 nephrectomy CKD mouse model with developing hypertension and atherogenesis 46. On the other hand, urate production is abundantly detected in microvascular endothelial cells and urate reduces endothelial nitric oxide 47, 48, 49, a crucial protective molecule from vascular injury. In addition, hyperuricemic rats have impaired endothelial function which can be reversed by lowering urate 50. Clinically, it has been shown that CKD stage 3A alone may not be a strong risk factor for cardiovascular events without HU, while dramatically elevated the risk accompanied by HU, indicating HU is a major determinant of increased CVD risk in CKD stage 3A 51. Taken together, our results tend to suggest a direct toxicity of urate to endothelial cells rather than secondary effects of CKD is not sufficient to induce atherosclerosis but may contribute to the neointimal lesions in perivascular carotid collar model.

It is important to note that there was only approximately 40% of survivors in the *Uox‐*KO birth cohort 10. Thus, the survived *Uox*‐KO mice with constant high urate levels may represent biased subjects and they normally develop significant metabolic and renal dysfunction, which actually mimics the human cases of gout and HU with severe complications. A transient increase in uricosuria during the first 2 weeks of life in *Uox*‐deficient mice might be responsible for the relative low survival rate 52. The current model is useful to study the asymptomatic HU from a metabolic perspective though it is not fully understood the reasons of poor survival rate. There is phenotypic heterogeneity existed between sexes in the *Uox*‐KO model. To avoid confounding factor due to gender or sex hormone difference, we only chose male mice in the current study. Further investigation in females would help to delineate a full picture of the effect of HU on cardiovascular diseases.

In conclusion, this is the first evidence to demonstrate that urate plays a contributing rather than a causal role in the carotid neointimal lesions, while ULT may bring additional benefits in this spontaneous HU male mouse model. Clinical and mechanism studies are warranted to investigate the ULT's anti‐atherosclerotic benefits in atherosclerosis patients with HU.

## Materials and methods

### Mouse model

The spontaneous HU mice were developed by knock‐out of the hepatically expressed *Uox* gene 10. Controls were their WT littermates on the C57BL/6J background. As previously described 53, we generated perivascular carotid collar placement mouse models with non‐occlusive silastic collars (length, 5 mm; internal diameter, 0.3 mm; external diameter, 0.6 mm) in 10‐week‐old males. Four weeks before surgery, the animals received high‐fat, high‐cholesterol (HF/HC) western‐type diets (21% (wt/wt) fat and 0.15% cholesterol). Mice were anesthetized by ketamine/xylazine and kept on continuous anesthesia during the surgery. Subsequently, a midline neck incision was made to surgically expose the right carotid artery, and the collar was positioned around the right carotid artery and held in place with a nylon sleeve. Both carotid sheaths were openned, and the common carotid arteries were dissected free from the surrounding connective tissue. The carotid arteries were then returned to their original position and the wound was sutured. After a 6‐week collar placement with western‐type diet, all mice were sacrificed for subsequent measurements. To evaluate the urate‐lowering effect, mice were gavaged with allopurinol at a dose of 100 mg·kg^−1^ from 6 to 16 weeks of age with collar induction at 10‐week‐old.

Mice were housed under specific pathogen free conditions at 22 °C under a 12‐h light/dark photoperiod with *ad libitum* access to rodent diet and sterile water 54. The Animal Research Ethics Committee of the Affiliated Hospital of Qingdao University approved this study.

### Blood biochemistry

Mice were fasted overnight before blood collection from the outer canthus. All biochemical indicators including SU, BUN, serum creatinine, fasting glucose, lipid profiles [TC, triglyceride (TG), high‐ and low‐ density lipoprotein cholesterol (HDL‐C, LDL‐C) were measured by an automatic biochemical analyser (Toshiba, Tokyo, Japan).

### Elisa

Plasma MCP‐1, ICAM‐1 and VCAM‐1 levels were quantified using an ELISA kit (R&D Systems, Minneapolis, MN, USA).

### Quantitative real‐time RT‐PCR

Total RNA was isolated from the carotid artery or cell line using trizol reagent (Roche Pharmaceuticals, Basel, Switzerland) and then reverse transcribed using a Fast Quant RT kit (Takara‐Bio, Shiga, Japan), followed by amplification using primers for MCP‐1, ICAM‐1 and VCAM‐1 under the condition: 95 °C for 10 min and 40 cycles of 95 °C for 15 s, 58 °C for 20 s and 68 °C for 20 s. The threshold cycle (*C*_t_) was determined and used to calculate Δ*C*_T_ values. The ΔΔ*C*_t_ ($2^{-\Delta C_{t}}$ ) was used to calculate relative mRNA expression, with each measurement performed in triplicate. Primer sequences are shown in Table 3.

**Table 3:** Primers of quantitative real‐time RT‐PCR

Primers	(5′‐sequences‐3′)
h‐MCP‐1_F	GATGCAATCAATGCCCCAGTC
h‐MCP‐1_R	TCCTTGGCCACAATGGTCTTG
h‐ICAM‐1_F	GGCTGGAGCTGTTTGAGAAC
h‐ICAM‐1_R	TCACACTGACTGAGGCCTTG
h‐VCAM‐1_F	TCCCTACCATTGAAGATACTGGAAA
h‐VCAM‐1_R	GCTGACCAAGACGGTTGTATCTC
h‐β‐actin_F	CGCAAAGACCTGTACGCCAAC
h‐β‐actin_R	CACGGAGTACTTGCGCTCAGG

### Western blot

Protein was extracted from the carotid artery. Fifty microgram protein lysates were loaded and transferred to nitrocellulose membranes. Blots were incubated with the primary antibodies (1 : 2000; Abcam, Cambridge, UK) against MCP‐1 (Catalog no. ab25124), ICAM‐1 (Catalog no. ab119871), VCAM‐1 (Catalog no. ab134047) and GAPDH (Catalog no. ab8245) at 4 °C overnight. After incubation with horseradish peroxidase‐conjugated goat anti‐rabbit secondary antibodies, visualization was performed with an enhanced chemiluminescence kit (Thermo Scientific, Waltham, MA, USA). Each measurement was performed in triplicate.

### Echocardiographic analysis

Echocardiography was performed after anesthesia with isoflurane using a Vevo 2100 ultrasound system equipped with a MS400 probe (VisualSonics, Toronto, ON, Canada) for *in vivo* transthoracic ultrasound imaging. The heart was imaged in a two‐dimensional mode in the parasternal long‐axis view. An M‐mode cursor was positioned perpendicular to the interventricular septum and the posterior wall of the left ventricle at the level of the papillary muscles. Stroke volume was calculated as (LV Vol; d − LV Vol; s) and cardiac output as [(LV Vol; d − LV Vol; s) * HR]/1000, where, LV Vol d: end‐diastolic left ventricle volume; LV Vol s: end‐systolic left ventricle volume.

### Blood pressure

Systolic and DBP blood pressure was measured by the CODA programmable noninvasive tail‐cuff sphygmomanometer (Kent Scientific, Torrington, CT, USA). Mice underwent an acclimation period of seven consecutive days to the sphygmomanometer before experiments. MBP was calculated as [DBP + 1/3 (SBP − DBP)].

### Assessment of endothelium‐dependent vasodilatation

Vasorelaxation of isolated aortic ring segments were determined in oxygenated Kreb's solution. Aortic rings were precontracted with 0.1 μmol·L^−1^ noradrenaline after an equilibration period of 60 min. Dilation at each acetylcholine (0.001, 0.01, 0.1, 1.0, 10 μmol·L^−1^) concentration was measured and expressed as the percentage in response to noradrenaline.

### Pathology analysis and immunohistochemistry

Carotid arteries were removed and fixed in formalin followed by paraffin embedding of 5 μm serial sections. Tissue serial sections were incubated with anti‐mouse PCNA (1 : 50; Santa Cruz, CA, USA, Catalog no. sc25280) and anti‐mouse F4/80 (macrophage‐specific marker, 1 : 50; Santa Cruz, Catalog no. sc52664) rabbit polyclonal antibodies. Images were captured by Nikon Eclipse TE2000‐S microscope (Nikon, Chiyoda, Japan) and analysed by image‐pro plus software (version 6.0; Houston, TX, USA).

### Reactive oxygen species (ROS) measurement

Samples were incubated with 2 μmol·L^−1^ DHE fluorescence probe (Thermo Scientific) for 30 min at 37 °C in the dark to measure ROS levels. Fluorescence was determined using a Nikon 90i (Nikon) with excitation wavelength at 480 nm and emission wavelength at 610 nm.

### Soluble urate

As previously described 55, soluble urate was prepared by dissolving uric acid (UA; Sigma, St. Louis, MO, USA) in warmed media containing 1 m NaOH. The solution was tested to be free of mycoplasma, endotoxin and filtered before use. Crystals were not detectable under polarizing microscopy, nor did they develop during cell incubation.

### Cell culture

Human umbilical vein endothelial cells (Cell bank of Chinese Academy of Sciences) were cultured in human endothelial cell‐specific growth medium C‐22010 (PromoCell, Heidelberg, Germany) with 10% fetal bovine serum, 100 units·mL^−1^ penicillin and 100 μg·mL^−1^ streptomycin (Invitrogen, Waltham, MA, USA). The viability of HUVECs was measured by Cell Counting Kit‐8 (Beyotime Institute of Biotechnology, Shanghai, China) according to the manufacturer's instruction. Cells (10^5^/well) were plated in 96‐well plates and co‐incubated with soluble urate (200–800 μmol·L^−1^) after 1 mmol·L^−1^ probenecid (an organic anion transport inhibitor that blocks UA entry into cells) or 10 μmol·L^−1^ NAC (a ROS scavenger) intervention for 12 h.

### Statistical analysis

All statistical analyses were performed using graphpad prism software (version 7; San Diego, CA, USA). Data were presented as the mean ± SEM. Differences between groups were analyzed by Student's *t* test or one‐way analysis of variance followed by Newman–Keuls multiple comparison test as appropriate. *P* < 0.05 was considered to be statistically significant.

## Conflict of interest

The authors declare no conflict of interest.

## Author contributions

All authors approved the final version to be published. JL, MS, XW and CL design the study. JL, XY, ZL, XQ and XJ performed the experiments. JL, XW and CL analysed and interpreted the data. Prof. Li had full access to all of the data in the study and takes responsibility for the integrity of the data and the accuracy of the data analysis.
